# Identification of genes related to salt stress tolerance using intron-length polymorphic markers, association mapping and virus-induced gene silencing in cotton

**DOI:** 10.1038/s41598-017-00617-7

**Published:** 2017-04-03

**Authors:** Caiping Cai, Shuang Wu, Erli Niu, Chaoze Cheng, Wangzhen Guo

**Affiliations:** State Key Laboratory of Crop Genetics & Germplasm Enhancement, Hybrid Cotton R&D Engineering Research Center, Ministry of Education, Nanjing Agricultural University, Nanjing, 210095 China

## Abstract

Intron length polymorphisms (ILPs), a type of gene-based functional marker, could themselves be related to the particular traits. Here, we developed a genome-wide cotton ILPs based on orthologs annotation from two sequenced diploid species, A-genome *Gossypium arboreum* and D-genome *G*. *raimondii*. We identified 10,180 putative ILP markers from 5,021 orthologous genes. Among these, 535 ILP markers from 9 gene families related to stress were selected for experimental verification. Polymorphic rates were 72.71% between *G*. *arboreum* and *G*. *raimondii* and 36.45% between *G*. *hirsutum* acc. TM-1 and *G*. *barbadense* cv. Hai7124. Furthermore, 14 polymorphic ILP markers were detected in 264 *G*. *hirsutum* accessions. Coupled with previous simple sequence repeats (SSRs) evaluations and salt tolerance assays from the same individuals, we found a total of 25 marker-trait associations involved in nine ILPs. The nine genes, temporally named as *C1* to *C9*, showed the various expressions in different organs and tissues, and five genes (*C3*, *C4*, *C5*, *C7* and *C9*) were significantly upregulated after salt treatment. We verified that the five genes play important roles in salt tolerance. Particularly, silencing of *C4* (encodes WRKY DNA-binding protein) and *C9* (encodes Mitogen-activated protein kinase) can significantly enhance cotton susceptibility to salt stress.

## Introduction

In modern crop breeding, many traits of interest such as yield, quality, and resistance to biotic or abiotic stress need to be improved simultaneously to allow crops to survive in extreme environmental conditions and to safeguard the safety of crops. Since the majority of traits are complex and controlled by many genomic loci, each of which have small effects, molecular markers are the foundation for genomics-based crop improvement. Several types of molecular markers, such as restriction fragment length polymorphisms (RFLPs), sequence-characterized amplified regions (SCARs), simple sequence repeats (SSRs) and single nucleotide polymorphisms (SNPs), have been successfully used in molecular marker assisted-selection (MAS) in cotton^1, 2^, rice^3^, and wheat^4^. Functional markers (FMs) are a type of gene-based marker that was developed from sequence polymorphisms present in allelic variants of a functional gene at a given locus. FMs accurately discriminate between traits associated with alleles of a target gene, and are ideal molecular markers for MAS in breeding^5^. EST-SSRs (eSSRs), SSRs developed from expressed sequence tags (ESTs), and gene-based SNP markers, in which the discovery of SNPs is specific to candidate genes or transcript sequences, are the most widely used types of FMs in crop species^6, 7^.

As more genome information becomes available for crop species, intron-spanning markers have become an important type of FM and their development has greatly increased. Compared with exons, introns contain more variations due to a lower selection pressure during the evolutionary process. Intron length polymorphisms (ILPs) are the easiest identified molecular markers in introns. They can be conveniently detected by polymerase chain reaction (PCR), using primers designed for flanking exons. This technique is known as exon-primed intron-crossing PCR amplification (EPIC-PCR)^8^. The development of ILP markers is unique since they are gene-specific, co-dominant, hypervariable, neutral, convenient, and reliable. At first, ILP markers were used in a small number of crops, such as Medicago^9^, foxtail millet^10^, maize^11^, potato, and *Solanum nigrum* ^12^, and are restricted to a small number of genes. With more genome information released, Yang *et al*.^13^ conducted a more comprehensive study and extracted a total of 57,658 potential intron polymorphism (PIP) markers from 59 plant species, and created a web-based database of PIP markers (http://ibi.zju.edu.cn/pgl/pip/). The genome-wide development of ILP markers has also been reported in rice^14^ and foxtail millet^15^.

Cotton (*Gossypium* spp.) is the world’s most important natural textile fiber and is a significant oilseed crop. Four cultivated species have been independently domesticated: two tetraploids, *G*. *hirsutum* L. (AD)1 and *G*. *barbadense* L (AD)2, and two diploids, *G*. *herbaceum* L. (A1) and *G*. *arboreum* L. (A2)^16^. The ancestral A- and D-like genomes are thought to have diverged only 5–10 million years ago (MYA), and all allotetraploids were formed from interspecific hybridization events between an A-genome-like ancestral African species and a D-genome-like North American species 1–2 MYA^17^. Recently, the availability of data on the whole-genome of *Gossypium* in different cotton species, including *G*. *raimondii* (D5)^18, 19^, *G*. *arboreum* (A2)^20^, *G*. *hirsutum* acc. TM-1 (AD1)^21, 22^ and *G*. *barbadense* acc. 3–79 and Xinhai21 (AD2)^23, 24^, made it possible to develop cotton ILP markers at a genome-wide level. To date, no genome-wide exploitation of ILP markers has been reported in cotton. To achieve this, we screened for differences in the intron-lengths of orthologous A- and D-genome genes, which have a relatively high level of similarity, by comparing genome sequences and annotation information from *G*. *raimondii* and *G*. *arboreum*, and developed a large number of gene-based ILP markers. We selected a set of ILP markers representative of genes associated with abiotic stress response pathways to experimentally validate the levels of diversity between *G*. *arboreum* and *G*. *raimondii* or between two allotetraploid cultivated species (*G*. *hirsutum* acc. TM-1 and *G*. *barbadense* cv. Hai7124). Further, nine candidate ILP markers related to salt stress were confirmed by both multiple comparison and association mapping approaches using a set of natural Upland cotton accessions. We investigated the temporal and spatial expression profiles of the nine candidate genes associated with salt stress traits in different tissues and in response to salt stress treatment, and verified that the functional roles of five genes that are significantly induced by salt stress treatment by virus-induced gene silencing (VIGS) analysis. Our study not only provided genome-wide, gene-based ILPs marker resources in cotton, but also mined effectively the genes with salt-tolerance for developing abiotic-resistance cultivars in future cotton-breeding programs.

## Results

### Genome-wide comparison of orthologs between diploid and tetraploid cotton species

*G*. *raimondii* and *G*. *arboreum* genome annotation files were obtained from http://www.phytozome.net and http://cgp.genomics.org.cn, respectively, and were used to obtain the corresponding intron distributions. In the 37,505 protein-coding genes in the D-genome species, the number of introns was between 0 and 78, with 9,535 genes (25.42%) having no introns. In the 41,330 genes in the A-genome species, the number of introns was between 0 and 76, with 12,083 genes (29.23%) having no introns. The number and distribution of introns in the A- and D-genome cotton species is shown in Fig. S1.

The BLAST program was used to obtain A- and D-genome orthologous genes. In total, 9,598 genes from the D-genome were found to be highly homologous with 9,686 genes from the A-genome, according to the following rules: A-CDS/D-CDS ≥95%, A-mRNA/D-mRNA ≥80%. Using 9,598 genes from *G*. *raimondii* as probes, 2,528 gene sequences showed no differences between the A- and D-genome introns, and 44,966 introns from 7,070 genes showed at least one intron difference for each pair of orthologs, with the different intron-lengths ranging from 1 bp to 1,339 bp. Of these 44,966 introns, 13,683 (30.43%) had intron-length differences ranging from 10 bp to 1000 bp, and were used for further ILP marker development.

From an evolutionary point, one gene in the diploid *G*. *raimondii* correspond to one homologous gene in *G*. *arboreum* and two homeologs from the A and D subgenomes in tetraploid cotton species. So we investigated the distribution of the 7,070 genes in tetraploid cotton species using the whole genome sequence of *G*. *hirsutum* acc. TM-1^21^ and *G*. *barbadense* acc. 3–79^23^. In total, 6989 and 7001 genes were found to exist homologous gene in the A and D subgenomes in *G*. *hirsutum*, respectively; and 6806 and 6821 genes were found to exist homologous genes in the A and D subgenomes in *G*. *barbadense*, respectively (Dataset S1), indicating more than 94% of 7070 genes can be used for ILP marker detection in tetraploid cotton species.

**Figure 1:**
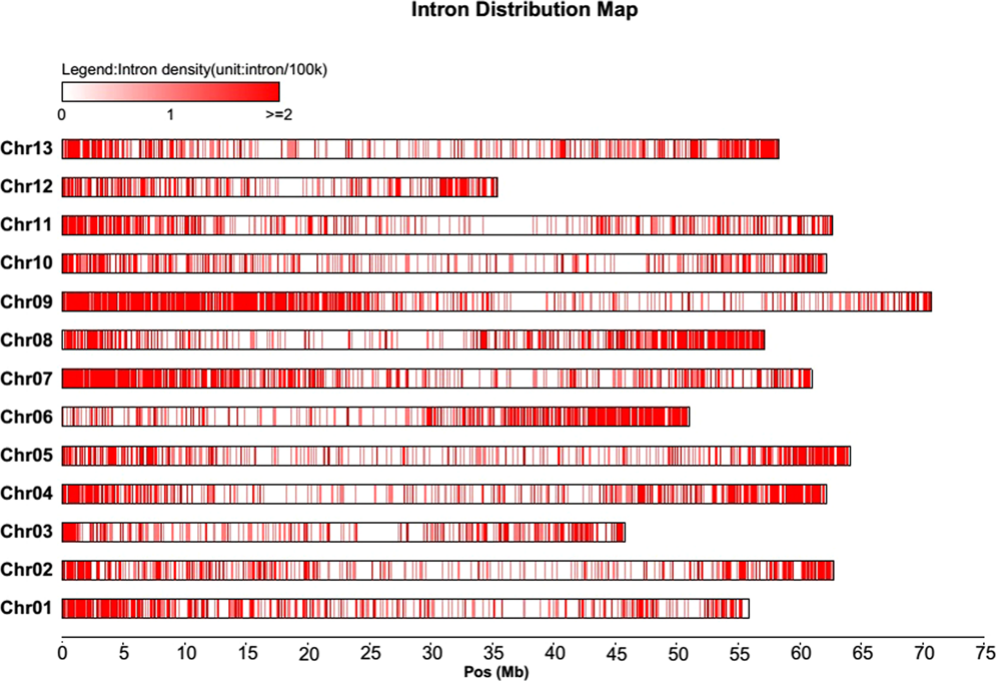
Chromosome distribution of 7,049 genes associated with ILP markers in *G*. *raimondii*. Each vertical short bar indicates the position of corresponding candidate gene.

Based on the genome of the diploid cotton species *G*. *raimondii* ^18^, we investigated the physical location of the 7,070 genes with intron-length differences between the A- and D-genome orthologs. In total, 7,049 genes were mapped to 13 chromosomes, with an average gene density of 9.408 genes/Mb, and the remaining 21 genes were mapped to the 8 scaffolds. Chromosome distribution showed that the highest frequency of these genes were found on Chr. 9, which contained 935 genes (935/7049, 13.264%) and had 13.222 genes/Mb, followed by Chr. 7 (708 genes, 10.044%, 11.610 genes/Mb). The lowest average gene density was found on Chr. 10 (7.382 genes/Mb), and the lowest frequency of genes on Chr. 12 (331 genes, 4.696%) (Fig. 1; Table 1). These genes were unevenly distributed across each chromosome with an increasing density towards one end of the chromosome on Chr. 1, 6, 7, 8, and 9, and both ends on Chr. 2, 3, 4, 5, 10, and 13 (Fig. 1).

**Table 1:** Chromosome distribution of developed ILP markers in *G*. *raimondii* genome.

Chr./Scaffold*	No. of genes	No. of genes developed markers	No. of primer pairs	Chromosome length (Mb)	Physical density (markers/Mb)%
Chr01	501	354	680	55.86823	8.967529
Chr02	501	357	719	62.76943	7.981592
Chr03	354	255	504	45.76565	7.735059
Chr04	575	392	806	62.17826	9.247605
Chr05	515	360	756	64.14041	8.029259
Chr06	557	397	799	51.07452	10.90563
Chr07	708	507	1056	60.98247	11.60989
Chr08	606	449	917	57.12882	10.60761
Chr09	935	693	1407	70.71302	13.22246
Chr10	459	329	678	62.17517	7.382368
Chr11	514	364	753	62.68101	8.200251
Chr12	331	224	462	35.42995	9.342379
Chr13	493	340	643	58.32116	8.453192
Scaffold	21	/	/	/	/
Total	7070	5021	10180	749.2281	9.40835

^*^The nomenclature of Chr./Scaffold in *G*. *raimondii* is from Paterson *et al*.^18^.

### Bioinfomatic analysis of orthologs with intron difference

The 7,070 genes were further subjected to functional annotation. Of these, 5,695 (80.552%) were mapped to 24,683 GO terms using Gene ontology annotation and were categorized into the three main GO classes (biological processes, molecular functions, and cellular components) (Dataset S2). In detail, the majority of the GO terms were grouped into metabolic process and cellular process categories within the biological processes, binding and catalytic categories within the molecular functions, and cell and cell part categories within the cellular components.

Using Kyoto Encyclopedia of Genes and Genomes (KEGG) analysis, 2,948 sequences from 1,332 genes (18.84%) were assigned to 135 different metabolic pathways (Dataset S3, Table 2). Of them, 95.828% of sequences (2,825) were mapped to the metabolism GO class, and the remaining 4.172% were assigned to genetic information processing (26, 0.882%), environmental information processing (53, 1.798%) and organismal systems (44, 1.493%) classes. The sequences in the metabolism GO class were largely involved in carbohydrate metabolism (770 clusters, 27.257%), amino acid metabolism (460 clusters, 16.283%), and lipid metabolism (348 clusters, 12.319%). In the categories of genetic information processing, environmental information processing, and organismal systems, sequences were further assigned to translation, signal transduction, and immune system subcategories, respectively. As a comparison, we also mapped 9,670 sequences from 4,441 genes in *G*. *raimondii* to 143 different metabolic pathways (Dataset S3). We found that there was a strong linear correlation (R^2^ = 0.986) between the annotated metabolic pathways of the 2,948 sequences from 1,332 genes and the 9,670 sequences from 4,441 *G*. *raimondii* genes (Table 2, Dataset S3). In different metabolic pathways, the genes with the highest number of intron differences were related to signal transduction (53/147, 36.05%), followed by energy metabolism (224/647, 34.62%), amino acid metabolism (460/1356, 33.92%), carbohydrate metabolism (770/2310, 33.33%), and then immune system processes (44/212, 20.75%).

**Table 2:** KEGG classification of 7,070 orthologs and their percentage in the whole genome.

Classification*	Numbers in 7,070 genes	Numbers in *G*. *raimondii* genome	Percentage (%)
1. Metabolism	2825	9225	30.62
1.1 Carbohydrate metabolism	770	2310	33.33
1.2 Energy metabolism	224	647	34.62
1.3 Lipid metabolism	348	1193	29.17
1.4 Nucleotide metabolism	187	673	27.79
1.5 Amino acid metabolism	460	1356	33.92
1.6 Metabolism of other amino acids	110	355	30.99
1.7 Glycan biosynthesis and metabolism	103	348	29.60
1.8 Metabolism of cofactors and vitamins	167	608	27.47
1.9 Metabolism of terpenoids and polyketides	88	296	29.73
1.10 Biosynthesis of other secondary metabolites	203	747	27.18
1.11 Xenobiotics biodegradation and metabolism	165	689	23.95
1.12 Chemical structure transformation maps	/	3	0.00
2. Genetic Information Processing	26	85	30.59
2.2 Translation	26	85	30.59
3. Environmental Information Processing	53	147	36.05
3.2 Signal transduction	53	147	36.05
4. Organismal Systems	44	212	20.75
4.1 Immune system	44	212	20.75

^*^KEGG annotation and metabolism maps were performed with BLAST2GO.

The rate of ILP variation was then investigated in nine gene families relevant to stress responses: NAC and WRKY transcription factors, mitogen-activated protein kinase (MAPK), heat shock proteins (HSPs), cytochrome P450 (CYP450), WD40 repeat-containing proteins (WD40s), Zinc finger (ZnF), leucine-rich repeat (LRR), and aquaporin family proteins. Lower correlation coefficient (R^2^ = 0.1436) was detected when the number of genes with ILPs were compared to all genes in the *G*. *raimondii* genome. The most highly conserved gene family was the leucine-rich repeat family, where there were only 66 members showed ILPs for A- and D-genome orthologs with total 1514 leucine-rich repeats in *G*. *raimondii* genome. The NAC family was the next most highly conserved (27/313), followed by the MAPK (12/128), WRKY (22/220), P450 (49/477), WD40 (65/531), heat shock protein (47/309), aquaporin (12/59), and zinc finger (133/385) families. The relationship between the sequence variations and functional diversity among orthologs remains to be investigated.

### Development and identification of ILP markers in cotton

The exon-primed intron-crossing PCR (EPIC-PCR) method^8^ was used to amplify intron polymorphisms. Based on bioinformatic analysis, we selected introns between 50 and 1000 bp in length with an intron length difference of 10–1000 bp, to develop ILP primers. Taking the D-genome sequence as reference and selecting polymorphic introns with at least 100 bp-length exons flanking the intronic region, a total of 10,180 ILP primers from 5,021 genes were developed (Dataset S4). Furthermore, we synthesized 535 ILP markers derived from nine gene families relevant to stress response, for experimental validation. Of them, 14 ILP markers were associated with the aquaporin protein family, 24 with the heat shock protein family, 25 with the NAC family, 27 with the WRKY family, 37 with the MAPK family, 59 with the P450 family, 74 with the leucine-rich repeat kinase family, 111 with the WD40 family, and 164 with the zinc finger transcription factor family (Dataset S5).

Of these 535 ILP markers, 411 primer pairs (76.82%) produced desirable and stable amplification products between *G*. *herbaceum* var. *africanum* and *G*. *raimondii* diploid species, with 54 (10.09%) failures in *G*. *herbaceum* var. *africanum*, 19 (3.55%) failures in *G*. *raimondii*, and 51 (9.53%) failures in both cotton species. The PCR products amplified by 389 primer pairs showed polymorphisms in the two diploid species, presenting a polymorphic rate of 72.71%. The highest polymorphic rate was in the WRKY transcription factor family (24/27; 88.89%); followed by the NAC family (21/25; 84%), the WD40 family (84/111; 75.68%) and the leucine-rich repeat family (56/74; 75.68%). P450 family members had the lowest polymorphic rate (37/59; 62.71%) (Table 3, Dataset S5).

**Table 3:** ILP markers polymorphism between diploid A- and D-genome (A/D), or between tetraploid A_t_D_t_-genome (GhA_t_D_t_/GbA_t_D_t_) in nine tested gene family*.

Gene family	No. of tested marker	A/D		GhA_t_D_t_/GbA_t_D_t_	
		No. of polymorphic marker	Polymorphic rate (%)	No. of polymorphic marker	Polymorphic rate (%)
Aquaporin	14	10	71.43	9	64.29
Heat shock proteins	24	16	66.67	4	16.67
NAC transcription factors	25	21	84.00	6	24.00
WRKY transcription factors	27	24	88.89	15	55.56
Mitogen-activated protein kinase	37	26	70.27	12	32.43
Cytochrome P450	59	37	62.71	14	23.73
Leucine-rich repeat	74	56	75.68	29	39.19
WD40 repeat-containing proteins	111	84	75.68	40	36.04
Zinc finger	164	115	70.12	66	40.24
Total	535	389	72.71	195	36.45

*A/D means two diploid cotton species, *G*. *herbaceum* var. *africanum* (A-genome) and *G*. *raimondii* (D-genome).

GhA_t_D_t_/GbA_t_D_t_ means two allotetraploid cultivated species, *G*. *barbadense* cv. Hai7124 and *G*. *hirsutum* acc. TM-1.

**Figure 2:**
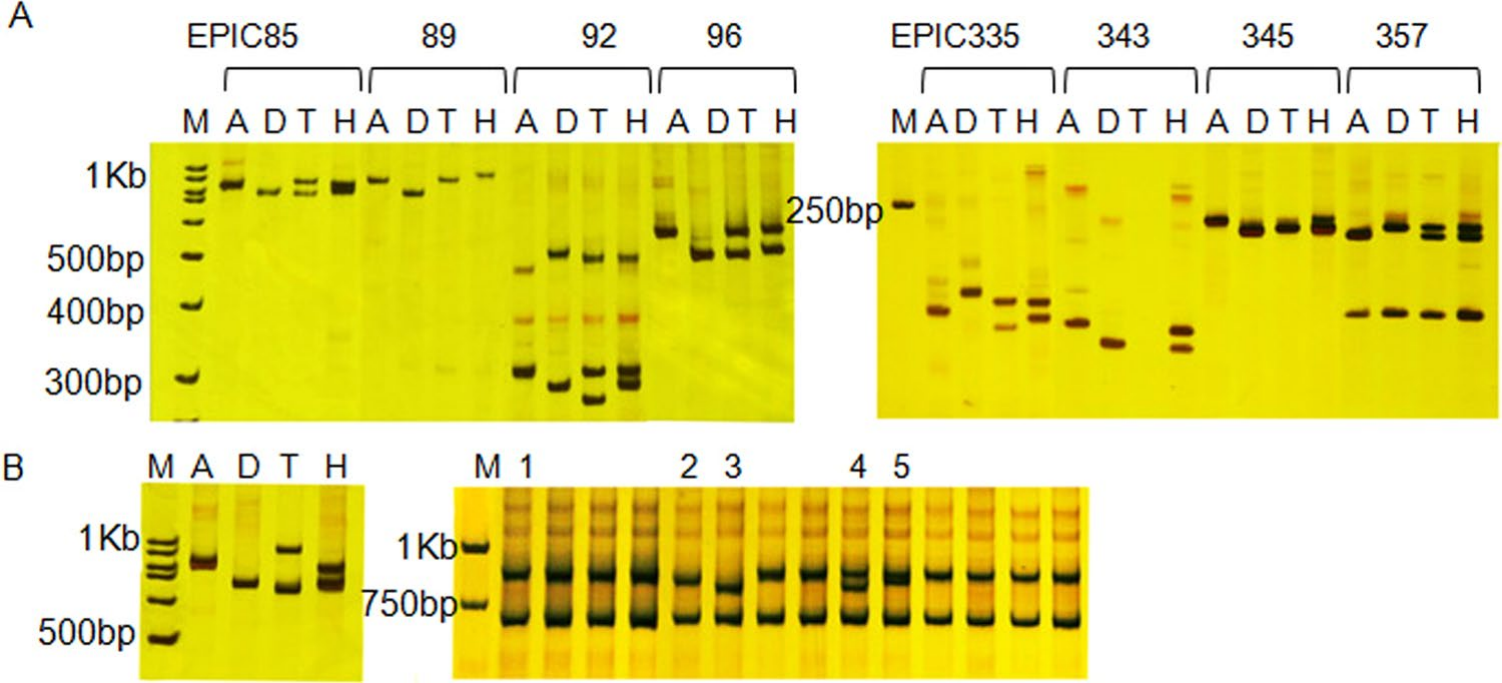
Electropherogram of detecting ILP markers polymorphism in cotton. (**A**) Electropherogram of ILP marker in four cotton species. M: marker; A: *G*. *herbaceum* var. *africanum*; D: *G*. *raimondii*; T: *G*. *hirsutum* acc. TM-1; H: *G*. *barbadense* cv. Hai7124. (**B**) Electropherogram of ILP marker EPIC211 in different *G*. *hirsutum* accessions. M: marker; A: *G*. *herbaceum* var. *africanum*; D: *G*. *raimondii*; T: *G*. *hirsutum* acc. TM-1; H: *G*. *barbadense* cv. Hai7124. Numbers 1–5 represents five alleles for EPIC211 detected in different *G*. *hirsutum* accessions.

We further used 535 ILP markers to screen allotetraploid interspecific polymorphisms of *G*. *hirsutum* acc. TM-1 and *G*. *barbadense* cv. Hai7124. Of these, 195 polymorphisms were detected, yielding a 36.45% polymorphic rate. We found that ILP markers associated with aquaporin, WRKY, zinc finger, leucine-rich repeat, and WD40 transcription factor families yielded the highest number of polymorphisms, with polymorphic rates of 64.29%, 55.56%, 40.24%, 39.19%, and 36.04%, respectively; while the heat shock protein family had the lowest polymorphic rate of 16.67%, with 4 of the tested markers found to be polymorphic (Table 3). As an example, an electropherogram of eight ILP markers (four WRKY and four leucine-rich repeat transcription factors) showed distinguishable A- and D-genome and/or At- and Dt-subgenome polymorphisms (Fig. 2A). The newly synthesized 535 ILP primer sequences, corresponding intron information, and data on polymorphisms between diploid cotton species *G*. *herbaceum* var. *africanum* and *G*. *raimondii*, and between tetraploid cultivated cotton species TM-1 and Hai7124, are presented in Dataset S5.

### Association analysis of salt stress traits in Upland cotton cultivars

Based on previous studies^25^, we investigated the ILPs from 264 *G*. *hirsutum* accessions. We firstly selected eight cotton varieties, derived from different germplasm pedigrees and three different ecological areas, to screen for polymorphic ILP markers. Polymorphisms were detected in 14 ILP markers and these were used to amplify alleles in the 264 accessions. In total, 41 alleles were detected, with 2.927 alleles per locus on average (ranging from two to five) and the highest number of alleles detected in EPIC211 (Fig. 2B). The average genetic diversity was 0.400 (ranging from 0.127 to 0.684) and the average polymorphism information content (PIC) was 0.345 (ranging from 0.122 to 0.623). Correlation between ILP markers and the ten salt stress tolerance traits, including relative germination rate (RGR) and germination percentage (RGP) at germinating stage, relative plant height (RPH), shoot dry matter (RSDM), root dry matter (RRDM), chlorophyll content (RCC), malondialdehyde (RMDA) content, and superoxide dismutase (RSOD), peroxidase (RPOD) and catalase (RCAT) enzyme activity at seedling stages, reported in Du *et al*.^25^, was assessed using multiple comparison and association analyses.

First, multiple comparisons were conducted to analyze the correlations between the ten salt stress traits and each polymorphic ILP marker using the LSR method in SPSS18.0. We found that there were 21 marker-trait correlations, involving 8 ILP markers and nine salt stress traits, showed significant differences between polymorphic loci and salt stress trait (P < 0.05). Of these, 13 marker-trait correlations reached statistical significance levels of P < 0.01 (Table S1).

Next, we analyzed the population structure of 264 *G*. *hirsutum* accessions using STRUCTURE V2.3.3 software based on 14 ILP markers and 145 SSR markers^25^. By comparing LnP (D) and ΔK, we selected K = 7 as the number of subpopulations, and corresponding Q-matrix data were used for the subsequent association mapping. The results showed that 18 marker-trait associations, involving 9 ILP markers and nine salt stress traits, were detected both from the MLM and GLM models (P < 0.05) (Table S2).

Integrated with the above two analysis, a total of 25 marker-trait associations involving 9 ILP markers for ten salt stress traits were detected (Table 4). Among these, two ILPs were from the WD40 family, two ILPs were from the leucine-rich repeat family, and the others were from MAPK, P450, WRKY, zinc finger, and aquaporin families, respectively. EPIC531, its gene encoding to plasma membrane intrinsic protein, was simultaneously associated with six salt stress traits: RCC, RPOD, RMDA, RPH, RRDM and RSDM. EPIC477, related to Zinc finger protein, was simultaneously associated with five salt stress traits: RCC, RPH, RSDM, RPOD, and RCAT. EPIC109 (Mitogen-activated protein kinase), EPIC309 (WD40 repeat-like protein) and EPIC356 (WRKY DNA-binding protein) were simultaneously associated with three salt stress traits. EPIC109 was associated with RCC, RPOD and RMDA; EPIC309 was associated with RCC, RPH and RPOD; and EPIC356 was associated with RSDM, RGR and RGP. EPIC50 (Leucine-rich repeat protein kinase family protein) was simultaneously associated with two salt stress traits (RRDM and RSDM). EPIC66 (Leucine-rich repeat protein kinase), EPIC211 (Cytochrome P450 protein) and EPIC274 (WD40 repeat-like protein) was associated with one salt stress trait each: RRDM, RPH and RMDA, respectively.

**Table 4:** Association information on ILP marker-traits related to salt stress.

Markers	Gene name	Gene ID	Sequence Description	Salt stress traits									
				RCC	RPH	RRDM	RSDM	RSOD	RPOD	RCAT	RMDA	RGR	RGP
EPIC50	*C1*	Gorai.005G026700	Leucine-rich repeat protein kinase family protein			MC^*^, AM^*^	MC^**^, AM^**^						
EPIC66	C2	Gorai.007G047200	Leucine-rich repeat protein kinase family protein			AM^*^							
EPIC274	C3	Gorai.006G261800	Transducin/WD40 repeat-like superfamily protein								MC^*^, AM^*^		
EPIC356	C4	Gorai.012G051500	WRKY DNA-binding protein 3					MC^*^				MC^**^, AM^**^	MC^**^, AM^**^
EPIC531	C5	Gorai.009G418100	Plasma membrane intrinsic protein 2	MC^*^, AM^*^	MC^**^	MC^*^		AM^*^	MC^**^, AM^*^		MC^**^, AM^**^		
EPIC309	C6	Gorai.010G020600	Transducin/WD40 repeat-like superfamily protein	MC^**^, AM^*^	AM^*^				MC^*^				
EPIC211	C7	Gorai.011G060100	Cytochrome P450 superfamily protein		MC^*^, AM^*^								
EPIC477	C8	Gorai.008G210800	Zinc finger (C3HC4-type RING finger) family protein	MC^**^, AM^*^	MC^**^, AM^*^		MC^**^		MC^**^	AM^*^			
EPIC109	C9	Gorai.003G139900	Mitogen-activated protein kinase 3	MC^*^					MC^**^, AM^*^		MC^**^, AM^*^		

^1^MC and AM means marker- trait associations detected in multiple comparisons and association mapping analysis, respectively.

^2^* and **: significant difference *P* < 0.05 and *P* < 0.01, respectively.

^3^Abbreviation for 10 traits related to salt tolerance is relative chlorophyll content (RCC), relative plant height (RPH), relative root dry matter (RRDM), relative shoot dry matter (RSDM), relative SOD activity (RSOD), relative POD activity (RPOD), relative CAT activity (RCAT) and relative MDA content (RMDA), relative germination rate (RGR), relative germination percentage (RGP), respectively.

### Potential function of candidate genes in cotton salt-tolerance

**Figure 3:**
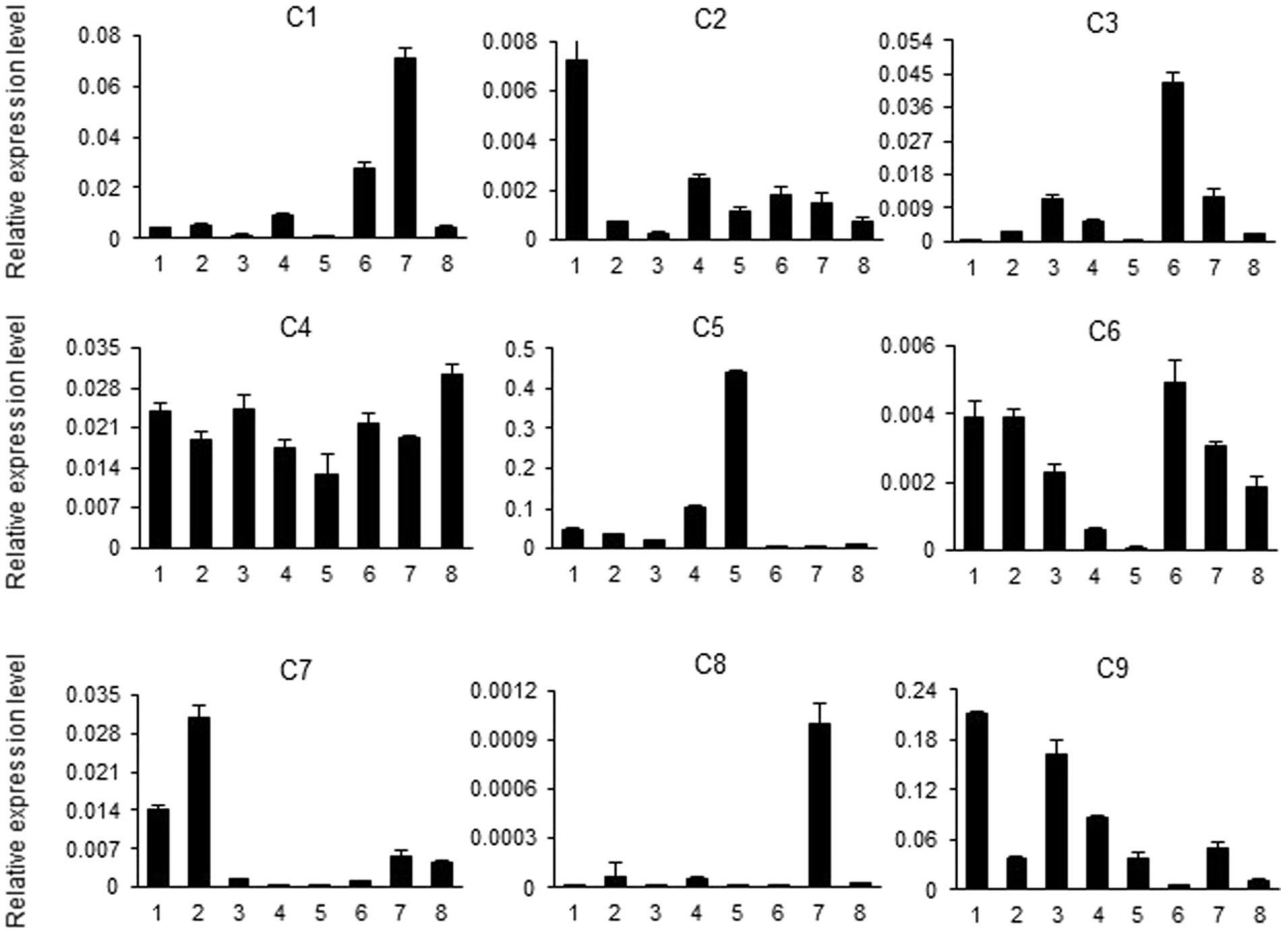
Expression patterns of nine candidate genes for salt-tolerance traits in different tissues and developmental stages. 1–8 indicate root, stem, leaf, petal, anther, 0 DPA ovule, 10 and 20 DPA fibers in TM-1, respectively. The error bars were calculated based on three biological replicates using standard deviation. The cotton *histone3* (AF024716) gene was used as the reference gene.

**Figure 4:**
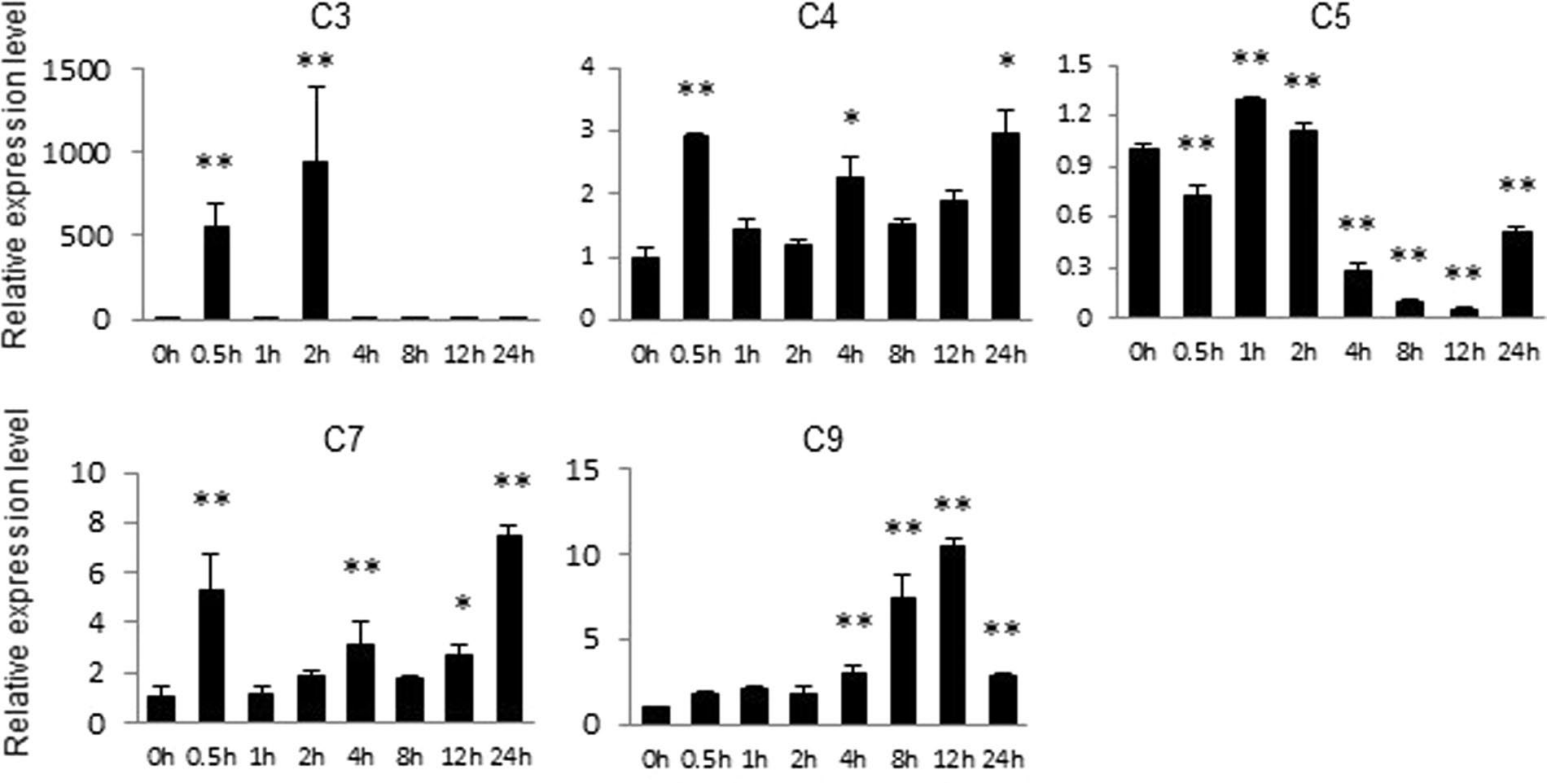
Expression analysis of five candidate genes induced after salt treatment. The error bars were calculated based on three biological replicates using standard deviation. The cotton *histone3* (AF024716) gene was used as the reference gene. “*” at *P* < 0.05, “**” at *P* < 0.01.

To confirm the relevance between the nine genes and salt tolerance, the salt induced expression patterns and functional characteristics based on VIGS analysis were investigated. We simplified to name the nine genes as *C1-C9* with their information described in Table 4. The expression patterns of the nine genes showed diverse expression patterns in different tissues and organs of *G*. *hirsutum* acc. TM-1, including roots, stems, leaves, petals, anthers, ovules and fibers at three different developmental stages (0 days post-anthesis [dpa], 10 dpa and 21 dpa) (Fig. 3). *C4* was a constitutively expressing gene in all tested tissues; *C6* had low expression in different tissues and organs, especially in petal and anther; *C9* was preferentially expressed in root and leaf, *C1* and *C8* were preferentially expressed in 10 DPA fibers; *C2*, *C3*, *C5* and *C7* was preferentially expressed in root, 0 DPA ovule, anther, stem, respectively. Then, we performed qRT-PCR to detect the differences in their expression abundance by 200 mM NaCl treatment in *G*. *hirsutum* cv. Jinmian 19. Five candidate genes (*C3*, *C4*, *C5*, *C7* and *C9*) were significantly induced after salt treatment (Fig. 4), with the highest expression for *C3* at 2 h, *C4* at 0.5 h, *C5* at 1 h, *C7* at 24 h, and *C9* at 12 h post treatment, respectively.

**Figure 5:**
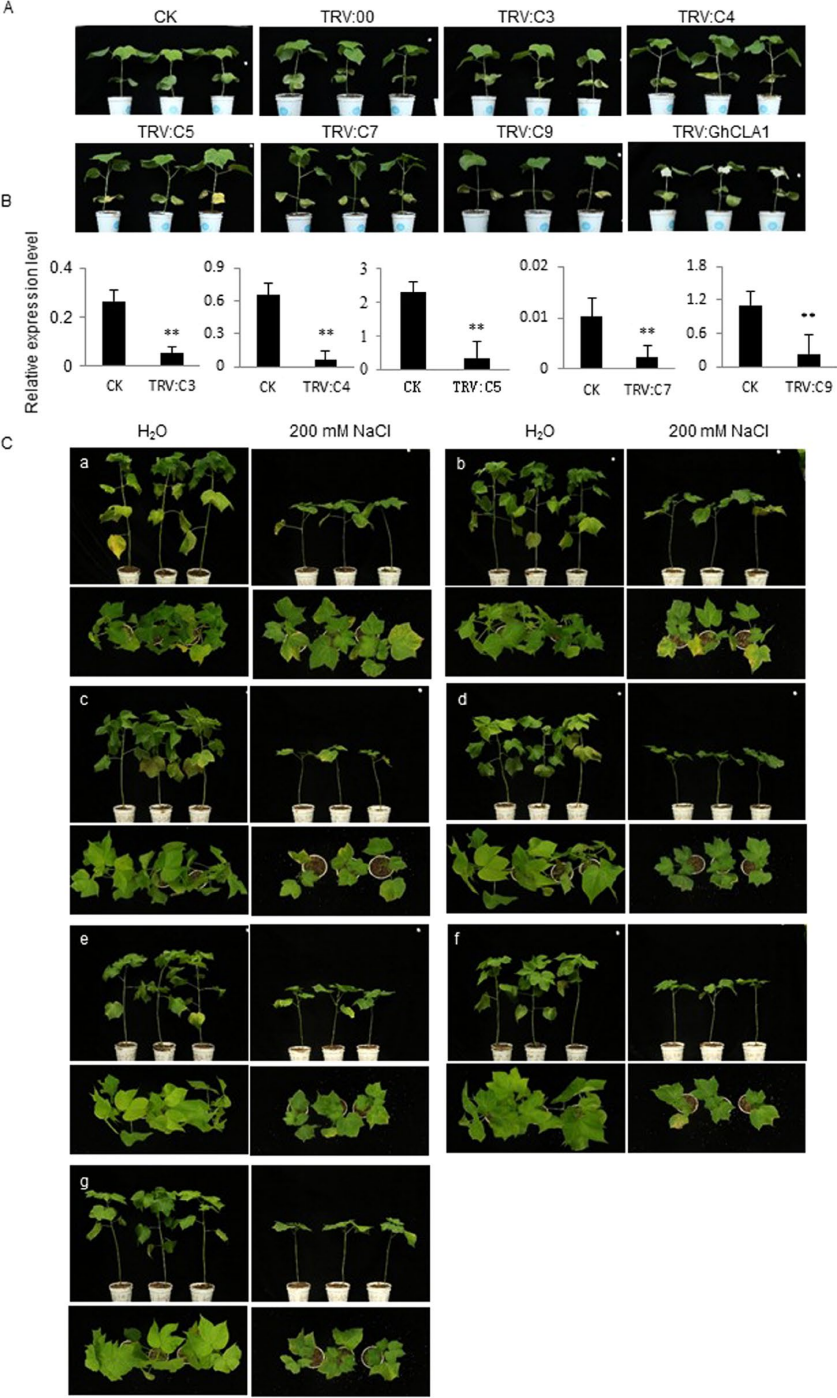
Plant phenotypes after target genes silencing with salt and mock treatments. (**A**) After two weeks infiltrated with TRV2: target silenced genes, the treated plants exhibited normal growth. TRV2:*GhCLA1* plants exhibited a photobleaching phenotype as indicator. TRV: 00 as the mock treatment and no-infiltration plants as CK. (**B**) Gene expression of five candidate genes in silenced and control plant leaves by real-time PCR analysis. The error bars were calculated based on three biological replicates using standard deviation. The cotton beta-tubulin gene (*GhTub1*) was used as the reference gene. “*” at *P* < 0.05, “**” at *P* < 0.01. (**C**) Phenotypes of target gene-silenced and control plants after the salt stress treatment. Plants were treated with 200 mM NaCl for a month (H_2_0 as control). a-g indicate CK (no-infiltration), TRV:00 (mock treatment), TRV:C3, TRV:*C4*, TRV:*C5*, TRV:*C7* and TRV:*C9*, respectively. Upper: photographed from plants side; Below: photographed from plants top.

To investigate the function of five candidate genes (*C3*, *C4*, *C5*, *C7* and *C9*) in salt stress tolerance, we constructed TRV2:*C3*, TRV2:*C4*, TRV2:*C5*, TRV2:*C7* and TRV2:*C9* vectors to silence the endogenous genes in TM-1 seedlings, with TRV:00 as the mock treatment and TRV2:*GhCLA1* (*Cloroplastos alterados* 1) as the technical control. More than 60 plants were infiltrated for each gene injection at 8 days post TM-1 seedlings. Two weeks later, the plants infiltrated with TRV2:*GhCLA1* displayed a photobleached phenotype (Fig. 5A). Real-time PCR showed that the no-infiltration plants (CK) appeared high expression levels of the corresponding target gene, however, the transcripts of the target gene exhibited strong silencing in TRV2: target silenced gene infiltrated plants (Fig. 5B).

**Figure 6:**
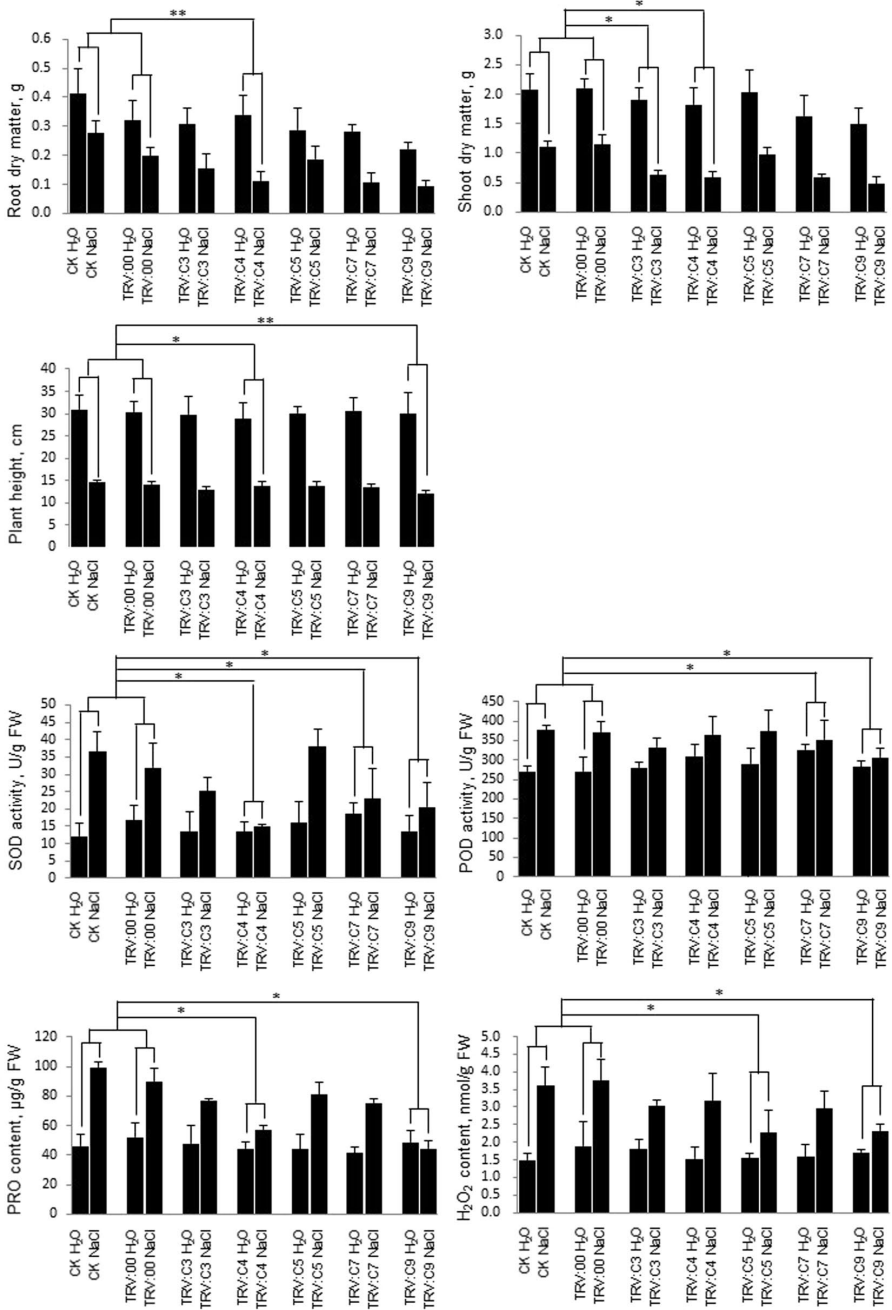
The difference of salt-tolerance parameters between gene-silenced and control plants in response to salt stress. Seven salt-tolerance parameters, including the plant height (cm), shoot dry matter weight (g), root dry matter weight (g), and SOD and POD (U/g FW) activity, and Pro (μg/g FW) and H_2_O_2_ (nmol/g FW) content were measured in leaves of gene-silenced and control plants with salt stress and water treatment. Plants were treated with 200 mM NaCl for a month (with water treatment as control). Significance level was compared between the difference between salt stress treatment and water control of target gene-silenced plants (TRV: target-silenced gene NaCl, and TRV: target-silenced gene H_2_O), and the difference between salt stress treatment and water control of no-infiltration plants (CK NaCl and CK H_2_O). “*”: at P < 0.05; “**”: at P < 0.01.

After the target gene was silenced, half of the plants per target gene-silenced plants were used for the salt stress treatment by irrigation 200 mM NaCl, and the others as water treatment control. A month later, there was no difference in growth between no-infiltration (CK), mock treated plants (TRV:00) and different target gene-silenced plant under tap water. However, cotton plants showed severe growth inhibition and lower true leaves defoliated under salt stress treatment. Especially, *C3*, *C4*, *C7* and *C9*-silenced plants exhibited a serious true leaves defoliated than *C5*, no-infiltration (CK) and mock treated plants (TRV:00) (Fig. 5C). Next, we measured seven traits related to salt tolerance for salt stress treatment and water treatment control plants. Compared salt stress treatment with water control plants, the plant height, shoot dry matter weight, and root dry matter weight was significantly decreased, and SOD and POD activity, and Pro and H_2_O_2_ content was drastically increased. We calculated the difference between salt stress treatment and water control of target gene-silenced plants (TRV: target-silenced gene NaCl, and TRV: target-silenced gene H_2_O), and the difference between salt stress treatment and water control of no-infiltration plants (CK NaCl and CK H_2_O), and compared the statistics significance between them. As shown in Fig. 6, the difference between TRV: *C4* NaCl and TRV: *C4* H_2_O for *C4*-silenced plants were more distinct in the plant height, shoot dry matter weight, root dry matter weight, SOD activity and Pro content than that between CK NaCl and CK H_2_O. Similarly, the significant difference in the plant height, SOD and POD activity, and Pro and H_2_O_2_ content was detected in *C9*-silenced plants after salt treatment. SOD and POD activity in *C7*-silenced plants, the shoot dry matter weight in *C3*-silenced plants, and the H_2_O_2_ content in *C5*-silenced plants were also significantly changed, respectively. Integrated the phenotype with physiological data, silencing of *C3*, *C4*, *C5*, *C7* and *C9* can compromise cotton salt tolerance in different extent, especially *C4* and *C9*, following *C7*, *C3*, and *C5*. The result indicated that increasing the gene expression for each have a potential utilization in cotton salt tolerance breeding.

## Discussion

Marker development in crop species is important in the facilitation of genomic-based crop improvement. In cotton, the most widely used type of DNA molecular markers is SSRs. Based on genome and EST sequence information from different *Gossypium* species, researchers have developed large quantities of SSR markers. In total, 17,448 publicly available SSRs have been deposited in the Cotton Database (http://www.cottondb.org/). These SSRs have been widely used in high-density genetic mapping^26–28^, target trait-related gene/QTL mapping^29–37^, association studies^25, 38–49^, and diversity analysis^50, 51^. As the large number of genomic sequence resources in *Gossypium* were released, the development of whole genome level markers, such as restriction-site associated DNA (RAD)^52, 53^ and insertion-deletion (InDel) and single nucleotide polymorphisms (SNPs)^7, 54–57^ has been initiated. FMs are a type of marker with the most potential to bridge the gap between structural polymorphisms and functional diversity, since these gene-derived markers are related to phenotypic variations. A pilot study for EST-based SSR, SNP, and InDel marker development and their utilization in tetraploid cotton genetic mapping has been carried out^6, 7^, however, the efficiency of marker development and the number of polymorphisms in designed primers were found to be relatively low using transcriptome data. High-throughput sequencing resources provide a better way to develop FMs in cotton. ILP markers are characterized as gene-specific, co-dominant, hypervariable, neutral, convenient, and reliable; however, to date, their genome-wide exploitation and application in cotton has not been reported. Here, we used whole-genome scaffold sequence information from the diploid cottons *G*. *raimondii* ^18^ and *G*. *arboreum* ^20^ to mine conserved orthologous sequences and develop functional ILP markers of orthologs between the A- and D-genomes. A total of 10,180 ILP markers from 5,021 orthologs were developed, and the polymorphism of 535 ILP markers associated with nine classes of gene family relevant to stress responses was validated experimentally. As a result, polymorphic rates of 72.71% and 36.45% between A/D-genome diploids and between A_t_D_t_ tetraploid genome species were detected, respectively, implying the high efficiency of FM marker development using whole-genome scaffold sequence information.

The cotton lineage experienced an abrupt five- to sixfold ploidy increase approximately 60 MYA, shortly after its divergence from *Theobroma cacao*, and then an allopolyploidy event that reunited divergent *Gossypium* genomes approximately 1–2 MYA conferred a 30–36-fold duplication of ancestral angiosperm (flowering plant) genes in the cotton tetraploids *G*. *hirsutum* and *G*. *barbadense* ^18^. Whole genome duplication events brought about a substantial gene family expansion, and a larger number of paralogs (including tandem and segmental duplications) were generated. In the evolutionary process, these paralogs were with conserved domains but undergone various structural variations and functional diversity. In this study, we first verified orthologs between the A- and D-genomes for ILP markers development, and carried out *in silico* PCR analysis with strict no mismatch to confirm the primer specificity. As a result, of 10,180 ILP markers from 7,070 genes, 9,294 and 9,713 ILPs were mapped in At- and Dt-subgenome of TM-1 genome, respectively, and 94.35% (8,769) and 97.05% (9,426) were uniquely matched (Dataset S4). The result indicated that these ILP markers corresponded to a particular gene with few interference from paralogous genes, and could be used to verify the homologs polymorphism in tetraploid cotton species. This study also provides a reference for the development and application of whole-genome ILP FMs in other complex polyploid organisms.

Biotic and abiotic stresses, such as *Verticillium dahliae*, drought, heat, submergence, and high salinity, can severely affect plant growth and crop productivity, which leads to worldwide economic losses. To understand and improve stress responses and tolerances in crop species, researchers have focused on the signaling perception mechanisms, transcriptional regulation and expression of functional proteins in the stress response^58^. Previous studies found that NAC transcription factors are involved in plant development and biotic and abiotic stress regulation^59^; WRKY transcription factors are important components of many aspects in the plant defense systems and in plant abiotic stress responses^60^; HSPs, which were originally identified as heat-inducible gene products, are a family of highly conserved proteins that respond to a wide variety of stresses including oxidative and thermal stress^61^; CYP450 proteins function as growth and developmental signals and protect plants from various biotic and abiotic stresses^62^; WD40s play a key role in protein-protein and protein-DNA interactions by acting as scaffolding molecules, promoting protein activity and responsiveness to salt stress^63^; ZnF and LRR family proteins play regulatory roles in immune responses^64, 65^; aquaporins regulate the movement of water and other small molecules across plant vacuolar and plasma membranes, and are associated with plant tolerance of biotic and abiotic stresses^66^; MAPK cascade is one of the universal signaling pathways involved in responses to external stimuli, which play a crucial role in plant growth and development as well as biotic and abiotic stress responses^67^. In this study, we selected 535 ILP markers derived from these nine gene families that are related to stress responses for structural and functional identification.

In cotton, association analysis has been widely used in mining QTLs/genes related to the important agronomic traits, such as fiber qualities^38–43, 46^, yield and its components^41, 44, 45, 48^, salt stress^25, 68^, *Verticillium* wilt resistance^47^ and seed oil and protein contents^50^ etc. Nevertheless, further experiments need to be done to mine the genes and verify their function based on these marker-trait associations. In this study, we detected a total of 25 marker-trait associations involving 9 ILP markers for ten salt stress traits. Following these ILPs corresponding to genes, we found that five genes (*C3*, *C4*, *C5*, *C7* and *C9*) played important roles in cotton salt tolerance through TRV-VIGS analysis. Taken together, the ILP markers associated with the traits with interest will accelerate the findings of functional genes and utilization in breeding program.

In this study, the functional role of *C4* and *C9* in salt stress were further confirmed in cotton, which were in line with previous evidence. *C4* (EPIC356, gene ID: Gorai.012G051500 of *G*. *raimondii*), corresponding to *WKY18* in *G*. *raimondii* ^60^, which encodes WRKY DNA-binding protein, was simultaneously associated with RGR and RGP in this study. Our previous study reported that under salt or drought treatment, *WRKY18* expression levels were significantly increased, with a peak at 8 h of treatment with NaCl (200 mmol L^−1^), and at 8 h of treatment with PEG6000 (20%), indicating its role in abiotic stress tolerance^60^. *C9* (EPIC109, gene ID: Gorai.003G139900 of *G*. *raimondii*), corresponding to *MPK9* in *G*. *raimondii* ^67^, which encodes mitogen-activated protein kinase, was simultaneously associated with RPOD and RMDA. Zhang *et al*.^67^ found that *MPK9* was constitutively expressed at high levels in both vegetative and reproductive organs. *MPK9* can be induced by multi-stressors such as jasmonis acid, H_2_O_2_, salicylic acid, 4 °C, 37 °C, and wounding, and plays a role in plant defense responses and multiple stress-signaling pathways.

In summary, genome-wide ILP markers are powerful functional markers to bridge the gap between structural polymorphisms and functional diversity. The new gene-based FMs will be a useful resource for gene mining and breeding improvement for traits of interest via MAS in cotton. The methodology can also serve as a useful model for the development of FMs in other complex plant genomes. Further, five genes (*C3*, *C4*, *C5*, *C7* and *C9*) were verified to be related to salt stress tolerance and have potential to improve salt tolerance in cotton abiotic-resistance breeding.

## Materials and Methods

### Development of putative ILP markers

**Figure 7:**
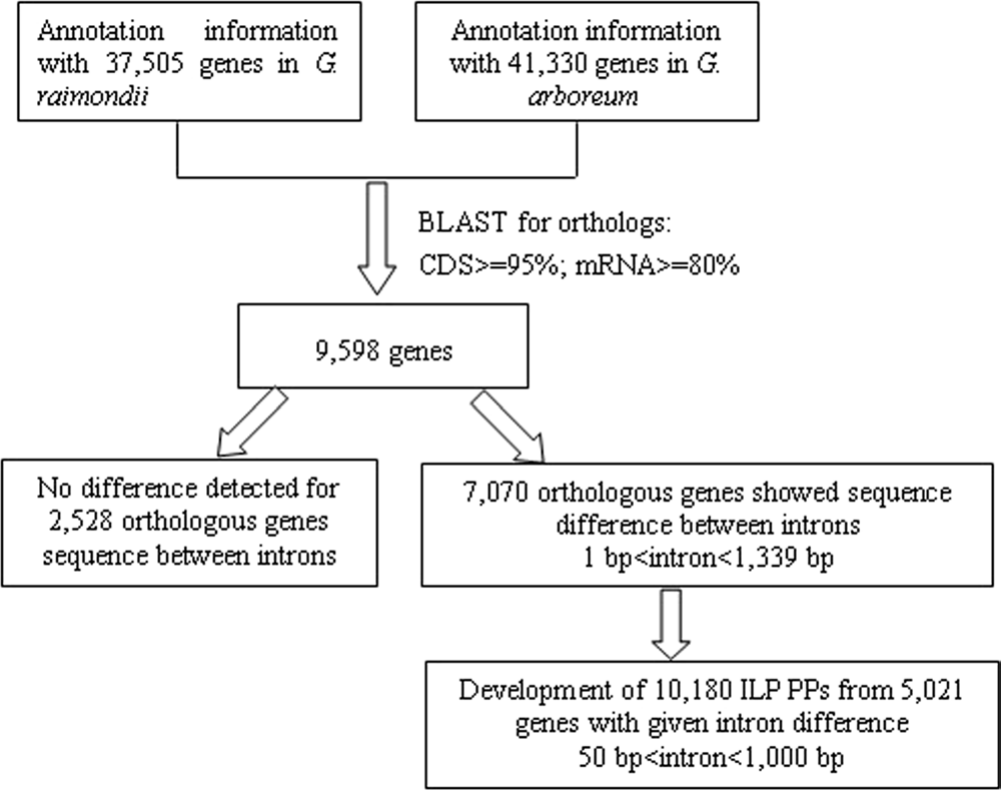
The development scheme of genome-wide ILP markers in this study.

The assembled genome sequences, annotated genes, transcripts and coding DNA sequences (CDSs) data were obtained from the genome annotation files for *G*. *raimondii* (http://www.phytozome.net) and *G*. *arboreum* (http://cgp.genomics.org.cn). The complete nuclear genome of 37,505 protein-coding genes in *G*. *raimondii* and 41,330 protein-coding genes in *G*. *arboreum* were extracted to identify conserved exons of single-copy genes. We extracted introns of a manageable size (<100 00 bp) as well as the corresponding flanking exons for further study. To detect orthologous genes in the two genomes, one-to-one orthologous relationship of genes between *G*. *arboreum* and *G*. *raimondii* were identified using reciprocal BLAST best hit with a high *E-value* (≥10^−20^). Further, transcripts and CDSs from the A genome were mapped to the D genome, and transcripts with an mRNA similarity ≥80% and a CDS similarity ≥95% were considered as orthologs. Orthologs with intron lengths between 50 and 1000 bp were investigated, and those with an intron length difference ranging from 10 bp to 1000 bp were used for the development of ILP orthologs primers. The scheme of ILP marker development is described in Fig. 7.

Primer pairs were designed from the flanking exon sequences using Primer3 software (http://www-genome.wi.mit.edu/genome_sofware/other/primer3.html). Two perl scripts served as interface modules between MISA and Primer3; p3_in.pl for creating a primer3 input file which was submitted to Primer3, and p3_out.pl, which was used to calculate and merge the information. These two perl scripts were downloaded from MISA (http://pgrc.ipk-gatersleben.de/misa/). To verify the primer specificity, *in silico* PCR analysis (parameter:gap 0-mismach 0-product size 50–2000) was employed to examine the uniqueness of the primer in different cotton species. The primer pairs for experimental validation were synthesized by GenScript (Nanjing, China). All primer information, including the primer design parameters, is shown in Dataset S4.

After obtaining Gene Ontology (GO) annotations for orthologs, Web Gene Ontology Annotation Plot (WEGO) software was used to carry out GO functional classification and to characterize the distribution of gene functions at the macro level^69^.

### Plant materials and treatments

A total of 267 cotton accessions for ILP marker analysis were collected for this study (Table S3). Firstly, two diploid cotton species, *G*. *herbaceum* var. *africanum* (A-genome) and *G*. *raimondii* (D-genome), were chosen for validating the efficiency of ILP markers. Secondly, two allotetraploid cultivated species (*G*. *barbadense* cv. Hai7124 and *G*. *hirsutum* acc. TM-1.), and 264 *G*. *hirsutum* germplasm accessions (including TM-1) were used for the analysis of ILP marker polymorphisms and their association with salt stress traits. Information on ten salt tolerance traits and 145 SSRs in 264 upland cotton cultivars was obtained from Du *et al*.^25^.

*G*. *hirsutum* L. acc TM-1, a genetic standard line of Upland cotton, was used for tissue/organ expression analysis. The plants were cultivated under normal field conditions. Petals and anthers were sampled on the day of flowering, and ovules and fibers were excised from developing flower buds or bolls on selected days post anthesis (dpa). Roots, stems and leaves were collected from two-week-old seedlings. The materials were quick-frozen in liquid nitrogen and stored at −70 °C before use.

*G*. *hirsutum* L. cv. Jinmian 19, which exhibits high tolerance to abiotic stress, was used for the salt stress treatments. Cotton seedlings (*G*. *hirsutum* L. cv. Jinmian 19) were grown in a growth chamber under greenhouse conditions at 28 °C under a 16 h light/8 h dark cycle for three weeks. Then the roots of cotton seedlings were irrigated with 200 mmol/L NaCl (ddH_2_O as a mock control). The leaves were harvested with three biological repeats at different time points (0, 0.5, 1, 2, 4, 8, 12 and 24 hours) after NaCl treatment, quick-frozen in liquid nitrogen and stored at −70 °C for RNA extraction.

### PCR amplification, RNA isolation and real-time PCR analysis

Genomic DNA from the cotton accessions involved in the study was isolated as described by Paterson *et al*.^70^. PCR reactions were carried out with 0.5 units of Taq DNA polymerase (Tiangen), 50–100 ng of template DNA, 1 μL of each primer (5 μM/μL), 0.2 μL of each dNTPs (10 mM), 0.6 μL MgCl_2_ (25 mM), and 1 μL of 10 × PCR reaction buffer in a final volume of 10 μL. PCR amplifications were performed using a Peltier Thermal Cycler-225 (MJ Research), and the amplification conditions were as follows: an initial denaturation at 95 °C for 5 minutes, followed by 28 cycles of 30 s at 94 °C, 30 s at 58 °C, and 30 s at 72 °C, and a final extension of 10 min at 72 °C. PCR products were separated using 9% polyacrylamide gel electrophoresis (PAGE) as described by Zhang *et al*.^71^.

Total RNA was extracted using plant total RNA extraction kit (Bioer, Hangzhou, China). 200–500 ng RNA samples were reverse transcribed into cDNA by HiScript reverse transcriptase (Vazyme, Nanjing, China). Based on the candidate gene sequences, gene-specific real-time PCR primers were designed using Beacon Designer 7.0. The predicted amplified fragment lengths were between 75 and 200 bp, and the annealing temperatures were between 58 °C and 60 °C. The amplification reactions of real-time PCR were performed on an ABI Prism 7500 (Applied Biosystems, USA) with the light cycler fast start DNA Master SYBR Green I kit (Roche, Basel, Switzerland) and three replicates. The amplification reactions were as follows: 95 °C for 10 min, followed by 40 cycles of 95 °C for 15 s, 58 °C for 15 s, and 72 °C for 30 s. The gene expression levels were calculated according to Livak and Schmittgen^72^. Two reference genes (*GhHis3* ^73^ with abundant transcripts and *GhTub1* ^74^ with moderate transcripts), were used simultaneously to confirm the real-time PCR results. The information for the real-time PCR primers were listed in Dataset S6.

### Association analysis

Multiple comparison and association mapping methods were used to confirm marker-trait association analysis. The multiple comparisons were estimated using SPSS18.0 (http://www.spss.com.cn/), and were conducted using the least significant range (LSR) method for correlation analysis of ten salt stress traits and different marker alleles. STRUCTURE v2.3.3 software^75^ (http://pritch.bsd.uchicago.edu/software.html) was used to infer the population structure of 264 *G*. *hirsutum* accessions (K = 1 to 10, with five runs at each K) using a burn-in of 10,000 and a run length of 100,000. The most likely number of clusters (K) was selected by comparing LnP (D) and ΔK^76^. A mixed linear model (MLM) and a general linear model (GLM) were employed to construct marker-trait association tests using the TASSEL 2.0.1 software package^77^.

### Virus induced gene silencing assays

*G*. *hirsutum* cv. TM-1 was used for VIGS analysis. The pTRV1 and pTRV2 vectors for VIGS analysis were generously provided by Dr. Libo Shan of Texas A & M University (College Station, TX, USA). *GhCLA1* (Cloroplastos alterados 1), which encoded 1-deoxy-D-xylulose-5-phosphate synthase, was constructed pTRV2:*GhCLA1* and acted as a control to verify the VIGS efficiency^78^. The primer pairs information used for the construction of VIGS vectors, the length of the segments of silence and corresponding base position are listed in Dataset S6. 201–430 bp PCR fragments for the corresponding target gene were amplified from TM-1 cDNA, double digested with *EcoRI* and *XbaI*, and inserted into enzyme sites for insertion into pTRV2 to make pTRV2 vectors with target silenced genes.

Agrobacterium were prepared and infiltrated as described by Cai *et al*.^79^. Eight-day-old TM-1 seedlings were infiltrated into two fully expanded cotyledons with a 1:1 mixture of Agrobacterium GV3101 carrying TRV1 and TRV2: target silenced gene. At the same time, cotton seedlings were infiltrated TRV1 and TRV2 or TRV1 and TRV2:*GhCLA1* as the mock treatment and the technical control, respectively. All cotton seedlings (including untreated plants) were kept at 23/22 °C (day/night) in a growth chamber with a 16 h light/8 h dark cycle for two weeks before they were used for identification of target gene silencing and next salt stress treatment. VIGS experiments were repeated with more than 60 plants for each different injection events per gene.

Total RNA was extracted and reverse transcribed into cDNA from candidate gene-silenced and untreated plants’ fresh leaf tissue two weeks post-inoculation by plant total RNA extraction kit (Bioer, Hangzhou, China) and HiScript reverse transcriptase (Vazyme, Nanjing, China), respectively. Real-time PCR was used to analyze the relative levels of candidate genes expression.

After candidate gene silencing, half of the plants (~30 plants) per candidate gene-silenced were used for the salt stress treatment by irrigation 200 mM NaCl, and the other 30 plants were irrigated tap water as a mock control. For salt and water treatment, each ~10 plants were investigated as a replicate. A month later, each plant was measured for the plant height, shoot dry matter weight, and root dry matter weight. Further, the superoxide dismutase and peroxidase enzyme activities, proline and H_2_O_2_ content were estimated using corresponding assay kit (Jiancheng, Nanjing, China). All indexes were detected with three biological replicates and three experimental replicates.

## Electronic supplementary material

Supplementary files

Dataset 1

Dataset 2

Dataset 3

Dataset 4

Dataset 5

Dataset 6
