# Supplementary material for: Identification of genes related to salt stress tolerance using intron-length polymorphic markers, association mapping and virus-induced gene silencing in cotton
Source: Sci Rep. 2017 Apr 3;7:528. doi: 10.1038/s41598-017-00617-7 (PMC5428780; doi:10.1038/s41598-017-00617-7)
Supplement: Supplementary file 1 — Supplementary files [file 41598_2017_617_MOESM1_ESM.pdf]

Identification of genes related to salt stress tolerance using intron-length polymorphic markers, association mapping and virus-induced gene silencing in cotton

Caiping Cai*, Shuang Wu*, Erli Niu, Chaoze Cheng, Wangzhen Guo**

State Key Laboratory of Crop Genetics & Germplasm Enhancement, Hybrid Cotton R & D Engineering Research Center, Ministry of Education, Nanjing Agricultural University, Nanjing 210095, China

* These authors contributed equally to this work.

** Correspondence should be addressed to Dr. Wangzhen Guo: State Key Laboratory of Crop Genetics & Germplasm Enhancement, Nanjing Agricultural University, Nanjing 210095, Jiangsu Province, P. R. China. E-mail: moelab@njau.edu.cn; Tel/Fax: +86 25 84396523

Supplementary Table S1: P-value of the multiple comparisons among 14 polymorphic ILP markers and ten salt stress traits using LSR method.

Marker	RCC	RPH	RRDM	RSDM	RSOD	RPOD	RCAT	RMDA	RGR	RGP
EPIC43	0.330189	0.2664683	0.140136	0.1265673	0.6400973	0.6532019	0.9503633	0.6908821	0.668473	0.2629496
EPIC50	0.9477636	0.2490145	0.0455406	0.0079397	0.9374364	0.5231868	0.4002706	0.5099329	0.2996398	0.3932581
EPIC66	0.2236345	0.9293267	0.0794249	0.4083292	0.1721954	0.9240334	0.1932283	0.3105367	0.7220842	0.4235914
EPIC93	0.0501571	0.4840041	0.7604592	0.9174137	0.669342	0.8465756	0.6911036	0.4439081	0.2866731	0.3915507
EPIC109	0.0461184	0.1013036	0.40708	0.43878	0.4571237	0.0009106	0.9886513	0.0059544	0.9252658	0.9011823
EPIC211	0.4809108	0.0260312	0.8449109	0.5201326	0.956907	0.4401007	0.3541233	0.565843	0.1633502	0.117503
EPIC274	0.5764312	0.5907478	0.0585886	0.0581699	0.5444198	0.9820513	0.6926915	0.0322182	0.1748746	0.1865865
EPIC309	0.0044862	0.1800835	0.814908	0.0850269	0.7562405	0.0227624	0.3400747	0.5513273	0.6877445	0.2890102
EPIC356	0.1263631	0.7392651	0.5717323	0.358869	0.0419185	0.6977786	0.8338784	0.5162895	0.0057297	0.0054428
EPIC369	0.194749	0.8782733	0.1537708	0.2316509	0.5919485	0.9406927	0.2702406	0.7294119	0.2555029	0.2370962
EPIC477	0.0002415	0.0003096	0.2226922	0.003155	0.0915398	0.0071576	0.3597189	0.0799785	0.8105475	0.3845179
EPIC525	0.256824	0.069049	0.977433	0.262719	0.132605	0.090342	0.479074	0.965627	0.793748	0.563122
EPIC526	0.548671	0.178552	0.45933	0.327821	0.224486	0.773206	0.233163	0.808144	0.574837	0.368094
EPIC531	0.010369	0.001165	0.021114	0.11808	0.633237	0.000252	0.599474	0.000872	0.101017	0.338966

Red indicates significant difference at P<0.05, and box in yellow filled means significant difference at P<0.01.

RCC:Relative Chlorophyll Content; RPH:Relative plant height; RRDM:Relative root dry matter; RSDM:Relative shoot dry matter; RSOD:Relative SOD activity; RPOD:Relative POD activity; RCAT:Relative CAT activity; RMDA:Relative MDA content; RGR:Relative germination rate; RGP:Relative germination percentage.

Supplementary Table S2: P-value and explained phenotypic variation of 18 marker-trait associations using MLM and GLM models.

Trait	Marker	p-MLM	r^2-MLM	p-GLM	r^2-GLM
RCC	EPIC309	0.0661	0.0193	0.0385	0.0264
RCC	EPIC477	0.0106	0.0379	0.0133	0.0376
RCC	EPIC531	0.0207	0.0461	0.0207	0.0461
RPH	EPIC211	0.0243	0.0414	0.0243	0.0414
RPH	EPIC309	0.0302	0.0241	0.0481	0.0224
RPH	EPIC477	0.0187	0.0321	0.0187	0.0322
RRDM	EPIC50	0.0438	0.0268	0.0472	0.0277
RRDM	EPIC66	0.0396	0.0208	0.029	0.0204
RSDM	EPIC50	0.0045	0.0377	0.0091	0.039
RSOD	EPIC531	0.0153	0.0525	0.0151	0.0531
RPOD	EPIC109	0.0265	0.0288	0.0265	0.0288
RPOD	EPIC531	0.0164	0.0458	0.0164	0.0458
RCAT	EPIC477	0.0308	0.0318	0.0308	0.0318
RMDA	EPIC109	0.0115	0.0408	0.0099	0.0428
RMDA	EPIC274	0.0153	0.0321	0.0221	0.0338
RMDA	EPIC531	9.187E-05	0.1007	6.715E-05	0.11
RGR	EPIC356	0.0086	0.0349	0.0086	0.0349
RGP	EPIC356	0.0096	0.0337	0.0094	0.034

**** p<0.01; * p<0.05**

RCC:Relative Chlorophyll Content; RPH:Relative plant height; RRDM:Relative root dry matter; RSDM:Relative shoot dry matter; RSOD:Relative SOD activity; RPOD:Relative POD activity; RCAT:Relative CAT activity; RMDA:Relative MDA content; RGR:Relative germination rate; RGP:Relative germination percentage.

Supplementary Table S3: Information of 267 tested accessions in this study.

No.	Species / Cultivar	Genomic group / Ecological area	Introduced or released year	Species
1	*G. herbaceum* var. *africanum*	A1	unknown	Wild diploid species
2	*G. raimondii*	D5	unknown	Wild diploid species
3	*G. barbadense* cv. Hai7124	AADD	unknown	Cultivated tetraploid sea island cotton accession
4	*G. hirsutum* acc. TM-1	AADD	unknown	Cultivated tetraploid upland cotton accession
5	*G. hirsutum* cv. Junmian1	Northwestern inland	1979	Cultivated tetraploid upland cotton accession
6	Sumian9	The Yangtze River	1995	Cultivated tetraploid upland cotton accession
7	Ejing1	The Yangtze River	1992	Cultivated tetraploid upland cotton accession
8	Daihongdai	The Yangtze River	1984	Cultivated tetraploid upland cotton accession
9	Simian3	The Yangtze River	1993	Cultivated tetraploid upland cotton accession
10	Esha28	The Yangtze River	1990	Cultivated tetraploid upland cotton accession
11	Sumian12	The Yangtze River	1997	Cultivated tetraploid upland cotton accession
12	Xiangmian16	The Yangtze River	1994	Cultivated tetraploid upland cotton accession
13	Simian2	The Yangtze River	1984	Cultivated tetraploid upland cotton accession
14	Xuzhou514	The Yangtze River	1990	Cultivated tetraploid upland cotton accession
15	52-128	The Yangtze River	1952	Cultivated tetraploid upland cotton accession
16	Emian14	The Yangtze River	1990	Cultivated tetraploid upland cotton accession
17	Yanmian48	The Yangtze River	1990	Cultivated tetraploid upland cotton accession
18	Sumian5	The Yangtze River	1993	Cultivated tetraploid upland cotton accession
19	Sumian3	The Yangtze River	1990	Cultivated tetraploid upland cotton accession
20	Xiangmian10	The Yangtze River	1982	Cultivated tetraploid upland cotton accession
21	Dongting1	The Yangtze River	1955	Cultivated tetraploid upland cotton accession
22	Chuanmian56	The Yangtze River	1992	Cultivated tetraploid upland cotton accession
23	Hua101	The Yangtze River	1992	Cultivated tetraploid upland cotton accession
24	Sumian2	The Yangtze River	1985	Cultivated tetraploid upland cotton accession
25	Ganmian8	The Yangtze River	1991	Cultivated tetraploid upland cotton accession
26	Ejing92	The Yangtze River	1990	Cultivated tetraploid upland cotton accession
27	Yanmian1	The Yangtze River	1987	Cultivated tetraploid upland cotton accession
28	Wanmian3	The Yangtze River	1978	Cultivated tetraploid upland cotton accession
29	Sumian4	The Yangtze River	1992	Cultivated tetraploid upland cotton accession

30	Sumian7	The Yangtze River	1994	Cultivated tetraploid upland cotton accession
31	Sumian8	The Yangtze River	1995	Cultivated tetraploid upland cotton accession
32	Sumian11	The Yangtze River	1997	Cultivated tetraploid upland cotton accession
33	Sumian15	The Yangtze River	1999	Cultivated tetraploid upland cotton accession
34	Sumian20	The Yangtze River	2002	Cultivated tetraploid upland cotton accession
35	Sumian22	The Yangtze River	2002	Cultivated tetraploid upland cotton accession
36	Simian1	The Yangtze River	1973	Cultivated tetraploid upland cotton accession
37	Siyang331	The Yangtze River	1982	Cultivated tetraploid upland cotton accession
38	Nantong5	The Yangtze River	1966	Cultivated tetraploid upland cotton accession
39	Humian204	The Yangtze River	1974	Cultivated tetraploid upland cotton accession
40	Eguangmian	The Yangtze River	1985	Cultivated tetraploid upland cotton accession
41	Emian4	The Yangtze River	1965	Cultivated tetraploid upland cotton accession
42	Emian6	The Yangtze River	1970	Cultivated tetraploid upland cotton accession
43	Emian7	The Yangtze River	1971	Cultivated tetraploid upland cotton accession
44	Emian9	The Yangtze River	1972	Cultivated tetraploid upland cotton accession
45	Emian12	The Yangtze River	1984	Cultivated tetraploid upland cotton accession
46	Emian13	The Yangtze River	1990	Cultivated tetraploid upland cotton accession
47	Emian17	The Yangtze River	1992	Cultivated tetraploid upland cotton accession
48	Emian18	The Yangtze River	1993	Cultivated tetraploid upland cotton accession
49	Emian20	The Yangtze River	1994	Cultivated tetraploid upland cotton accession
50	Emian21	The Yangtze River	1996	Cultivated tetraploid upland cotton accession
51	Emian23	The Yangtze River	2003	Cultivated tetraploid upland cotton accession
52	Ekangmian2	The Yangtze River	1993	Cultivated tetraploid upland cotton accession
53	Ekangmian3	The Yangtze River	1995	Cultivated tetraploid upland cotton accession
54	Ekangmian8	The Yangtze River	1998	Cultivated tetraploid upland cotton accession
55	Ekangmian9	The Yangtze River	1999	Cultivated tetraploid upland cotton accession
56	Ekangmian10	The Yangtze River	1999	Cultivated tetraploid upland cotton accession
57	Huakangmian1	The Yangtze River	2002	Cultivated tetraploid upland cotton accession
58	Yishuhong	The Yangtze River	1953	Cultivated tetraploid upland cotton accession
59	Xiangmian11	The Yangtze River	1985	Cultivated tetraploid upland cotton accession
60	Xiangmian12	The Yangtze River	1988	Cultivated tetraploid upland cotton accession
61	Xiangmian13	The Yangtze River	1989	Cultivated tetraploid upland cotton accession

62	Xiang4108	The Yangtze River	1984	Cultivated tetraploid upland cotton accession
63	Chuanmian30	The Yangtze River	1995	Cultivated tetraploid upland cotton accession
64	Chuanmian45	The Yangtze River	2000	Cultivated tetraploid upland cotton accession
65	Chuanmian239	The Yangtze River	1999	Cultivated tetraploid upland cotton accession
66	Shumian1	The Yangtze River	2000	Cultivated tetraploid upland cotton accession
67	Wanmian17	The Yangtze River	2000	Cultivated tetraploid upland cotton accession
68	Wanmian73-10	The Yangtze River	1985	Cultivated tetraploid upland cotton accession
69	Zhemian11	The Yangtze River	1997	Cultivated tetraploid upland cotton accession
70	Yumian1-1	The Yangtze River	2002	Cultivated tetraploid upland cotton accession
71	I4005	The Yangtze River	unknown	Cultivated tetraploid upland cotton accession
72	Yan1074	The Yangtze River	unknown	Cultivated tetraploid upland cotton accession
73	Xuzhou142	The Yellow River	1973	Cultivated tetraploid upland cotton accession
74	Jimian8	The Yellow River	1983	Cultivated tetraploid upland cotton accession
75	Lumian1	The Yellow River	1979	Cultivated tetraploid upland cotton accession
76	Zhongmiansuo3	The Yellow River	1960	Cultivated tetraploid upland cotton accession
77	Zhongmiansuo16	The Yellow River	1990	Cultivated tetraploid upland cotton accession
78	Jimian12	The Yellow River	1986	Cultivated tetraploid upland cotton accession
79	Shan1155	The Yellow River	1978	Cultivated tetraploid upland cotton accession
80	Shiyuan321	The Yellow River	1998	Cultivated tetraploid upland cotton accession
81	Zhongmiansuo12	The Yellow River	1989	Cultivated tetraploid upland cotton accession
82	Zhongmiansuo19	The Yellow River	1992	Cultivated tetraploid upland cotton accession
83	Zhongmiansuo23	The Yellow River	1995	Cultivated tetraploid upland cotton accession
84	Lumian6	The Yellow River	1984	Cultivated tetraploid upland cotton accession
85	Zhongmiansuo35	The Yellow River	1999	Cultivated tetraploid upland cotton accession
86	Zhongmiansuo41	The Yellow River	2002	Cultivated tetraploid upland cotton accession
87	Shiduan5	The Yellow River	1960	Cultivated tetraploid upland cotton accession
88	Zhongmiansuo5	The Yellow River	1971	Cultivated tetraploid upland cotton accession
89	Keyi2	The Yellow River	1970	Cultivated tetraploid upland cotton accession
90	Lumian2	The Yellow River	1983	Cultivated tetraploid upland cotton accession
91	Zhongmiansuo17	The Yellow River	1990	Cultivated tetraploid upland cotton accession
92	Jingsimian	The Yellow River	1942	Cultivated tetraploid upland cotton accession
93	Yumian1	The Yellow River	1981	Cultivated tetraploid upland cotton accession

94	86-1	The Yellow River	1975	Cultivated tetraploid upland cotton accession
95	Shan401	The Yellow River	1965	Cultivated tetraploid upland cotton accession
96	Jimian7	The Yellow River	1982	Cultivated tetraploid upland cotton accession
97	Zhongmiansuo45	The Yellow River	2003	Cultivated tetraploid upland cotton accession
98	Yumian5	The Yellow River	1989	Cultivated tetraploid upland cotton accession
99	Zhongmiansuo9	The Yellow River	1973	Cultivated tetraploid upland cotton accession
100	Zhongmiansuo24	The Yellow River	1997	Cultivated tetraploid upland cotton accession
101	Zhongmiansuo25	The Yellow River	1995	Cultivated tetraploid upland cotton accession
102	Zhongmiansuo27	The Yellow River	1998	Cultivated tetraploid upland cotton accession
103	Zhongmiansuo30	The Yellow River	1998	Cultivated tetraploid upland cotton accession
104	Zhongmiansuo40	The Yellow River	2002	Cultivated tetraploid upland cotton accession
105	Zhongmiansuo43	The Yellow River	2005	Cultivated tetraploid upland cotton accession
106	Zhongmiansuo44	The Yellow River	2004	Cultivated tetraploid upland cotton accession
107	Zhongmiansuo49	The Yellow River	2004	Cultivated tetraploid upland cotton accession
108	Zhongmiansuo50	The Yellow River	2005	Cultivated tetraploid upland cotton accession
109	Zhongzhimian2	The Yellow River	2005	Cultivated tetraploid upland cotton accession
110	Henan69	The Yellow River	1970	Cultivated tetraploid upland cotton accession
111	Yumian8	The Yellow River	1993	Cultivated tetraploid upland cotton accession
112	Yumian9	The Yellow River	1994	Cultivated tetraploid upland cotton accession
113	Yumian11	The Yellow River	1994	Cultivated tetraploid upland cotton accession
114	Yumian15	The Yellow River	1997	Cultivated tetraploid upland cotton accession
115	Yumian17	The Yellow River	1997	Cultivated tetraploid upland cotton accession
116	Yumian18	The Yellow River	1998	Cultivated tetraploid upland cotton accession
117	Yumian19	The Yellow River	1999	Cultivated tetraploid upland cotton accession
118	Yumian20	The Yellow River	1999	Cultivated tetraploid upland cotton accession
119	Yumian112	The Yellow River	2002	Cultivated tetraploid upland cotton accession
120	Yuzao73	The Yellow River	2002	Cultivated tetraploid upland cotton accession
121	Yu668	The Yellow River	2001	Cultivated tetraploid upland cotton accession
122	Jimian10	The Yellow River	1984	Cultivated tetraploid upland cotton accession
123	Jimian11	The Yellow River	1986	Cultivated tetraploid upland cotton accession
124	Jimian15	The Yellow River	1988	Cultivated tetraploid upland cotton accession
125	Jimian20	The Yellow River	1996	Cultivated tetraploid upland cotton accession

126	Jimian27	The Yellow River	1998	Cultivated tetraploid upland cotton accession
127	Jifeng106	The Yellow River	2005	Cultivated tetraploid upland cotton accession
128	Jihan3	The Yellow River	1970	Cultivated tetraploid upland cotton accession
129	sGK321	The Yellow River	2001	Cultivated tetraploid upland cotton accession
130	Handan109	The Yellow River	2002	Cultivated tetraploid upland cotton accession
131	GK99-1xuanxi	The Yellow River	2005	Cultivated tetraploid upland cotton accession
132	Han4849	The Yellow River	2004	Cultivated tetraploid upland cotton accession
133	Lumian9	The Yellow River	1990	Cultivated tetraploid upland cotton accession
134	Lumian10	The Yellow River	1990	Cultivated tetraploid upland cotton accession
135	Lumian11	The Yellow River	1992	Cultivated tetraploid upland cotton accession
136	Lumianyan16	The Yellow River	2005	Cultivated tetraploid upland cotton accession
137	Lumianyan18	The Yellow River	2005	Cultivated tetraploid upland cotton accession
138	Jinmian12	The Yellow River	1993	Cultivated tetraploid upland cotton accession
139	Jinmian21	The Yellow River	1997	Cultivated tetraploid upland cotton accession
140	Jinmian25	The Yellow River	1998	Cultivated tetraploid upland cotton accession
141	Jinmian28	The Yellow River	1999	Cultivated tetraploid upland cotton accession
142	Jinmian29	The Yellow River	2000	Cultivated tetraploid upland cotton accession
143	Jinmian36	The Yellow River	2003	Cultivated tetraploid upland cotton accession
144	Shanmian5	The Yellow River	1974	Cultivated tetraploid upland cotton accession
145	Shanmian6	The Yellow River	1975	Cultivated tetraploid upland cotton accession
146	Xuzhou58	The Yellow River	1972	Cultivated tetraploid upland cotton accession
147	Xuzhou209	The Yellow River	1955	Cultivated tetraploid upland cotton accession
148	Baimian1	The Yellow River	2004	Cultivated tetraploid upland cotton accession
149	Yinshan4	The Yellow River	2005	Cultivated tetraploid upland cotton accession
150	Zhengnongmian4	The Yellow River	2006	Cultivated tetraploid upland cotton accession
151	Yinshan6	The Yellow River	2006	Cultivated tetraploid upland cotton accession
152	sGK958	The Yellow River	2007	Cultivated tetraploid upland cotton accession
153	Yinshan7	The Yellow River	2008	Cultivated tetraploid upland cotton accession
154	Xinzhi5	The Yellow River	2008	Cultivated tetraploid upland cotton accession
155	Jinke178	The Yellow River	2009	Cultivated tetraploid upland cotton accession
156	Kelin9828	The Yellow River	2009	Cultivated tetraploid upland cotton accession
157	Jinkemian9	The Yellow River	2010	Cultivated tetraploid upland cotton accession

158	Xinmumian5	The Yellow River	2010	Cultivated tetraploid upland cotton accession
159	Yinshan8	The Yellow River	2010	Cultivated tetraploid upland cotton accession
160	Zhongmiansuo79	The Yellow River	2010	Cultivated tetraploid upland cotton accession
161	Fumian289	The Yellow River	2008	Cultivated tetraploid upland cotton accession
162	Aomian618	The Yellow River	2008	Cultivated tetraploid upland cotton accession
163	Baimian5	The Yellow River	2011	Cultivated tetraploid upland cotton accession
164	Baimian2	The Yellow River	2005	Cultivated tetraploid upland cotton accession
165	Jinxiumian1	The Yellow River	2010	Cultivated tetraploid upland cotton accession
166	Kekangmian2	The Yellow River	2010	Cultivated tetraploid upland cotton accession
167	Xinkang4	The Yellow River	2004	Cultivated tetraploid upland cotton accession
168	Handan885	The Yellow River	2005	Cultivated tetraploid upland cotton accession
169	Ji122	The Yellow River	2002	Cultivated tetraploid upland cotton accession
170	Han7860	The Yellow River	2005	Cultivated tetraploid upland cotton accession
171	Jimian616	The Yellow River	2003	Cultivated tetraploid upland cotton accession
172	Hanmian103	The Yellow River	2003	Cultivated tetraploid upland cotton accession
173	Han685	The Yellow River	2002	Cultivated tetraploid upland cotton accession
174	Jimian169	The Yellow River	2008	Cultivated tetraploid upland cotton accession
175	Ji151	The Yellow River	2003	Cultivated tetraploid upland cotton accession
176	Ji3927	The Yellow River	2008	Cultivated tetraploid upland cotton accession
177	Ji1516	The Yellow River	2003	Cultivated tetraploid upland cotton accession
178	Han5158	The Yellow River	2005	Cultivated tetraploid upland cotton accession
179	Hanmian559	The Yellow River	2008	Cultivated tetraploid upland cotton accession
180	Hanmian802	The Yellow River	2005	Cultivated tetraploid upland cotton accession
181	Jimian958	The Yellow River	2005	Cultivated tetraploid upland cotton accession
182	Guoxinmian9	The Yellow River	2009	Cultivated tetraploid upland cotton accession
183	Guoxinmian11	The Yellow River	2005	Cultivated tetraploid upland cotton accession
184	Chuangyoumian9	The Yellow River	2008	Cultivated tetraploid upland cotton accession
185	Lumianyan27	The Yellow River	2006	Cultivated tetraploid upland cotton accession
186	Shengmian1	The Yellow River	2008	Cultivated tetraploid upland cotton accession
187	Jinmian38	The Yellow River	2004	Cultivated tetraploid upland cotton accession
188	Lumianyan29	The Yellow River	2006	Cultivated tetraploid upland cotton accession
189	Lumianyan28	The Yellow River	2006	Cultivated tetraploid upland cotton accession

190	Guanmian4	The Yellow River	2008	Cultivated tetraploid upland cotton accession
191	Lumianyan32	The Yellow River	2008	Cultivated tetraploid upland cotton accession
192	sGK791	The Yellow River	2006	Cultivated tetraploid upland cotton accession
193	Lukenmian33	The Yellow River	unknown	Cultivated tetraploid upland cotton accession
194	Xinqiu4	The Yellow River	2009	Cultivated tetraploid upland cotton accession
195	Lumianyan36	The Yellow River	2009	Cultivated tetraploid upland cotton accession
196	Lumianyan37	The Yellow River	2009	Cultivated tetraploid upland cotton accession
197	Yinxingmian4	The Yellow River	2010	Cultivated tetraploid upland cotton accession
198	K638	The Yellow River	2010	Cultivated tetraploid upland cotton accession
199	Huamian5	The Yellow River	2010	Cultivated tetraploid upland cotton accession
200	Shannongmian8	The Yellow River	2010	Cultivated tetraploid upland cotton accession
201	Jimian228	The Yellow River	2005	Cultivated tetraploid upland cotton accession
202	Delinong5	The Yellow River	2010	Cultivated tetraploid upland cotton accession
203	Lumianyan22	The Yellow River	2004	Cultivated tetraploid upland cotton accession
204	Renhe39	The Yellow River	2009	Cultivated tetraploid upland cotton accession
205	Yinrui361	The Yellow River	2007	Cultivated tetraploid upland cotton accession
206	Xinqiu1	The Yellow River	2006	Cultivated tetraploid upland cotton accession
207	sGKmianxiang69	The Yellow River	2008	Cultivated tetraploid upland cotton accession
208	Zhengmian18	The Yellow River	2010	Cultivated tetraploid upland cotton accession
209	Liumian2	The Yellow River	2010	Cultivated tetraploid upland cotton accession
210	Jinmian13	The Yellow River	1993	Cultivated tetraploid upland cotton accession
211	Jinmian19	The Yellow River	1995	Cultivated tetraploid upland cotton accession
212	W-7	The Yellow River	unknown	Cultivated tetraploid upland cotton accession
213	W-5	The Yellow River	unknown	Cultivated tetraploid upland cotton accession
214	W-2	The Yellow River	unknown	Cultivated tetraploid upland cotton accession
215	W-3	The Yellow River	unknown	Cultivated tetraploid upland cotton accession
216	W-6	The Yellow River	unknown	Cultivated tetraploid upland cotton accession
217	W-8	The Yellow River	unknown	Cultivated tetraploid upland cotton accession
218	Jiaxing1	The Yellow River	1999	Cultivated tetraploid upland cotton accession
219	Lu668-4	The Yellow River	unknown	Cultivated tetraploid upland cotton accession
220	Lumianyan17	The Yellow River	2003	Cultivated tetraploid upland cotton accession
221	W-1	The Yellow River	unknown	Cultivated tetraploid upland cotton accession

222	W-4	The Yellow River	unknown	Cultivated tetraploid upland cotton accession
223	Zhongzhimian2xuanxi	The Yellow River	unknown	Cultivated tetraploid upland cotton accession
224	Xinluzao1	Northwestern inland	1991	Cultivated tetraploid upland cotton accession
225	Xinluzao3	Northwestern inland	1987	Cultivated tetraploid upland cotton accession
226	Xinluzao5	Northwestern inland	1994	Cultivated tetraploid upland cotton accession
227	Xinluzao7	Northwestern inland	1997	Cultivated tetraploid upland cotton accession
228	Xinluzao8	Northwestern inland	1997	Cultivated tetraploid upland cotton accession
229	Xinluzao9	Northwestern inland	1999	Cultivated tetraploid upland cotton accession
230	Xinluzao12	Northwestern inland	2000	Cultivated tetraploid upland cotton accession
231	Xinluzao13	Northwestern inland	2002	Cultivated tetraploid upland cotton accession
232	Xinluzao20	Northwestern inland	2005	Cultivated tetraploid upland cotton accession
233	Xinluzao22	Northwestern inland	2005	Cultivated tetraploid upland cotton accession
234	Xinluzao26	Northwestern inland	2005	Cultivated tetraploid upland cotton accession
235	Xinluzao28	Northwestern inland	2005	Cultivated tetraploid upland cotton accession
236	Xinluzao30	Northwestern inland	2005	Cultivated tetraploid upland cotton accession
237	Xinluzao32	Northwestern inland	2005	Cultivated tetraploid upland cotton accession
238	Xinluzhong2	Northwestern inland	1988	Cultivated tetraploid upland cotton accession
239	Xinluzhong8	Northwestern inland	1999	Cultivated tetraploid upland cotton accession
240	Xinluzhong21	Northwestern inland	2004	Cultivated tetraploid upland cotton accession
241	Xinluzhong33	Northwestern inland	2007	Cultivated tetraploid upland cotton accession
242	Xinluhzhong26	Northwestern inland	2006	Cultivated tetraploid upland cotton accession
243	Xinluzhong35	Northwestern inland	2007	Cultivated tetraploid upland cotton accession
244	Xinluzhong44	Northwestern inland	2010	Cultivated tetraploid upland cotton accession
245	Liaomian4	Northern (Specific early maturation)	1972	Cultivated tetraploid upland cotton accession
246	Guannong1	Northern (Specific early maturation)	1930	Cultivated tetraploid upland cotton accession
247	Heishanmian1	Northern (Specific early maturation)	1974	Cultivated tetraploid upland cotton accession
248	Liaomian1	Northern (Specific early maturation)	1959	Cultivated tetraploid upland cotton accession
249	Liaomian3	Northern (Specific early maturation)	1971	Cultivated tetraploid upland cotton accession
250	Liaomian6	Northern (Specific early maturation)	1980	Cultivated tetraploid upland cotton accession
251	Liaomian9	Northern (Specific early maturation)	1984	Cultivated tetraploid upland cotton accession
252	Liaomian13	Northern (Specific early maturation)	1994	Cultivated tetraploid upland cotton accession
253	Liaomian14	Northern (Specific early maturation)	1996	Cultivated tetraploid upland cotton accession

254	Liaomian16	Northern (Specific early maturation)	1999	Cultivated tetraploid upland cotton accession
255	Liaomian17	Northern (Specific early maturation)	2000	Cultivated tetraploid upland cotton accession
256	Liaomian19	Northern (Specific early maturation)	2003	Cultivated tetraploid upland cotton accession
257	Jinyu5	Northern (Specific early maturation)	1955	Cultivated tetraploid upland cotton accession
258	Jinmian1	Northern (Specific early maturation)	1958	Cultivated tetraploid upland cotton accession
259	Jinmian6	Northern (Specific early maturation)	1996	Cultivated tetraploid upland cotton accession
260	Chaoyangmian1	Northern (Specific early maturation)	1956	Cultivated tetraploid upland cotton accession
261	Foster6	from the US	1933	Cultivated tetraploid upland cotton accession
262	King	from the US	1890	Cultivated tetraploid upland cotton accession
263	DPL15	from the US	1950	Cultivated tetraploid upland cotton accession
264	Stoneville2B	from the US	1947	Cultivated tetraploid upland cotton accession
265	DPL16	from the US	1970	Cultivated tetraploid upland cotton accession
266	DP99B	from the US	unknown	Cultivated tetraploid upland cotton accession
267	NC20B	from the US	unknown	Cultivated tetraploid upland cotton accession

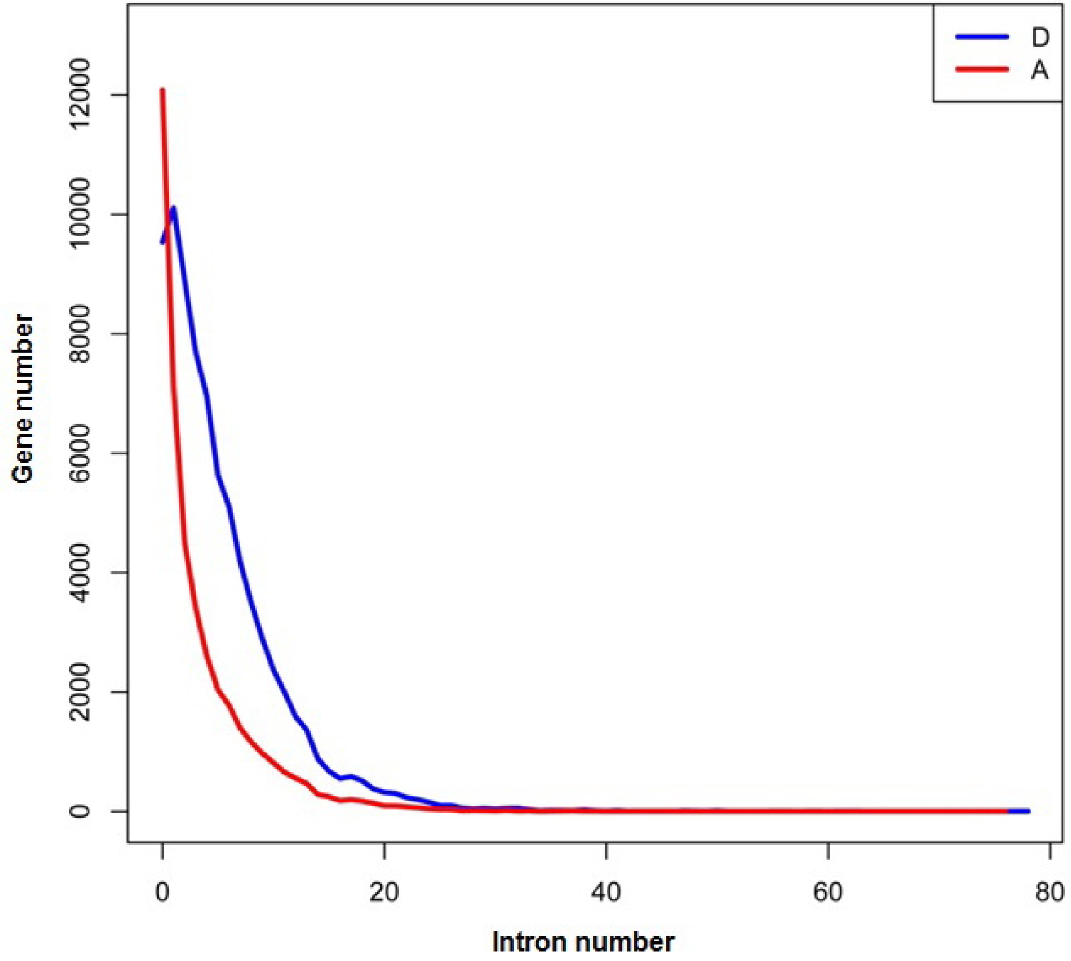

Supplementary Fig. S1: The number and distribution of introns in A- and D-genome cotton species.
